# Statistical Determination of the Gating Windows for Respiratory-Gated Radiotherapy Using a Visible Guiding System

**DOI:** 10.1371/journal.pone.0156357

**Published:** 2016-05-26

**Authors:** Se An Oh, Ji Woon Yea, Sung Kyu Kim

**Affiliations:** 1 Department of Radiation Oncology, Yeungnam University Medical Center, Daegu, Korea; 2 Institute of Medical Science, Yeungnam University College of Medicine, Daegu, Korea; 3 Department of Radiation Oncology, Yeungnam University College of Medicine, Daegu, Korea; North Shore Long Island Jewish Health System, UNITED STATES

## Abstract

Respiratory-gated radiation therapy (RGRT) is used to minimize the radiation dose to normal tissue in lung-cancer patients. Although determining the gating window in the respiratory phase of patients is important in RGRT, it is not easy. Our aim was to determine the optimal gating window when using a visible guiding system for RGRT. Between April and October 2014, the breathing signals of 23 lung-cancer patients were recorded with a real-time position management (RPM) respiratory gating system (Varian, USA). We performed statistical analysis with breathing signals to find the optimal gating window for guided breathing in RGRT. When we compared breathing signals before and after the breathing training, 19 of the 23 patients showed statistically significant differences (*p* < 0.05). The standard deviation of the respiration signals after breathing training was lowest for phases of 30%–70%. The results showed that the optimal gating window in RGRT is 40% (30%–70%) with respect to repeatability for breathing after respiration training with the visible guiding system. RGRT was performed with the RPM system to confirm the usefulness of the visible guiding system. The RPM system and our visible guiding system improve the respiratory regularity, which in turn should improve the accuracy and efficiency of RGRT.

## Introduction

The aim of radiation therapy is to provide a sufficient radiation dose to the tumors of cancer patients while minimizing the radiation dose to normal tissues [1]. Recently, various radiation-therapy techniques have been studied to achieve this goal, including intensity-modulated radiation therapy (IMRT), volumetric-modulated arc therapy (VMAT), and dynamic conformal arc therapy (DCAT) [2–6].

Reports from the Radiation Therapy Oncology Group (RTOG) [7–10] include guidelines for stereotactic body radiation therapy (SBRT) treatment of lung cancer and cover patient selection, techniques, dose fractions, dose verification at treatments, localization, simulations, immobilization, calculation algorithms, prescription dose constraints for treatment planning, and critical organ dose–volume limits. In particular, RTOG-0915 [10] suggests two methods for dealing with tumors with movements due to breathing: (1) If four-dimensional computed tomography (4DCT) is not applicable because of the tumor motion and patient condition, a conventional CT simulation (non-4DCT) is used in which the planned target volume (PTV) is defined by the gross target volume (GTV) with a 0.5-cm extension in the axial plane and 1.0-cm extension in the longitudinal plane. (2) If the 4DCT simulation is applicable, the internal target volume (ITV) generated by the tumor motion and derived from a 4DCT dataset is extended by 0.5 cm. Although adding large margins to the target volume helps deliver a sufficient radiation dose to the tumor, the radiation dose to the lung increases, which increases the chance of radiation pneumonitis [11, 12]. On the other hand, if the margin added to the target volume is too small, the uncertainty of the radiation dose to the target volume increases and leads to undesired treatment outcomes, although the radiation dose into the lung is decreased to reduce the chance of radiation pneumonitis.

Recent studies have focused on reducing the radiation dose to the lungs. In particular, the American Association of Physicists in Medicine (AAPM) Task Group 76 classified five types of strategies for managing motions caused by breathing during radiation therapy [13]: (1) the motion-encompassing method, (2) respiratory-gated techniques, (3) breath-hold techniques, (4) forced shallow-breathing methods, and (5) respiration-synchronized techniques.

A number of studies have considered respiratory-gated therapy, which enables treatment during specific phases of respiration by using an external marker block to receive signals in order to reduce the radiation dose to the lung while maintaining a sufficiently high radiation dose to tumors [14, 15]. Because a smaller gating window increases the treatment time while decreasing unnecessary radiation to the lungs, radiation oncologists generally randomly decide the gating window of a specific phase during which the beam is irradiated during gating treatment. At an inhalation phase of 0% and exhalation phase of 50%, gating windows are usually set at 30%–50%, 30%–60%, or 30%–70% [16–18].

Beddar et al. [19] investigated the correlation between the motions of an external marker and internal fiducials implanted in eight liver patients undergoing 4DCT. Their results showed a strong correlation between the external respiration and internal motion during expiration corresponding to the 40%–60% phases in the superior–inferior (SI) direction. They suggested that a real-time position management (RPM) trace is a reliable predictor for relating the internal marker tumor position and external marker during gating treatment.

In general, the external marker block motions differ from the internal tumor motions. Because surrogates such as diaphragms and injected fiducial markers have become more reliable, their use to confirm internal tumor motions has been suggested. Unfortunately, diaphragms and injected fiducial markers were not available for use at our institution. In our study, we only used the external marker block to determine the gating window for respiratory-gated radiotherapy (RGRT). In addition, we assumed that the correlation between the external marker block and internal tumor is intra-fractionally constant.

Analysis of the external marker signal is important in RGRT. However, there has not yet been any statistical study on using external marker blocks for determining the effective gating windows in gated radiotherapy. Therefore, the objective of our study was to identify the differences in breathing signals measured before and after breathing training by using RPM and our visible feedback system in RGRT and to determine the optimal gating window through a statistical analysis of the breathing signals after breathing training.

## Materials and Methods

### Ethics statement

**Fig 1:**
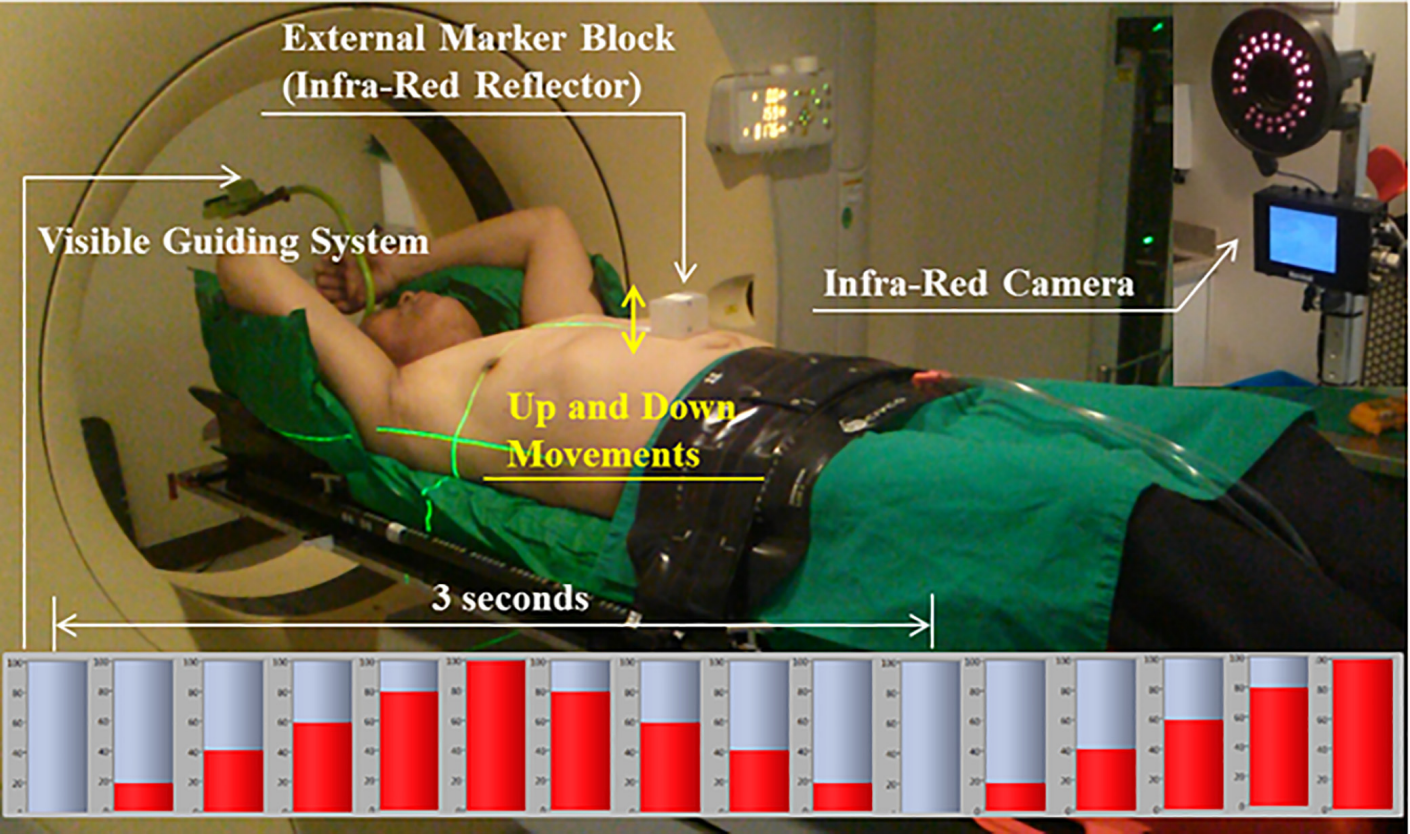
Setup for Recording the Breathing Signals of a Patient Using the Varian Real-time Position Management (RPM; Varian Medical Systems, Palo Alto, CA) System with a Visible Guiding System.

This study was approved by the Institutional Review Board (IRB) of Yeungnam University Medical Center (YUMC 2015-10-030); patient consent was specially waived because the patient data were investigated anonymously under the approval of the YUMC IRB. The individual pictured in Fig 1 has given written informed consent (as outlined in the PLOS consent form) to publish these case details.

### Patient selection

Lung-cancer patients who could undergo breathing training from April 29, 2014, to October 6, 2014, were included in the study. Table 1 lists the patient characteristics, including the gender, age, tumor site, and treatment techniques. There were 19 males and 4 females, and the average age was 67 years (ranging from 45 years to 85 years). Tumors were located in the right upper lobe (RUL) in nine patients, the right lower lobe (RLL) in six patients, the left upper lobe (LUL) in eight patients, and the left lower lobe (LLL) in one patient. Patient #21 had treatment to the RUL and the LUL. Patients underwent conventional radiotherapy (CRT) with a multi-fractionated 2- or 1.8-Gy dose and SBRT with a total dose of 12–15 Gy in four fractions.

**Table 1:** Characteristics of the Patients Included in the Study. Patient 21 Was Treated in the RUL and LUL.

Patient	Gender	Age	Tumor site	Techniques used for treatment
**1**	M	59	RLL	SBRT
**2**	M	74	LUL	SBRT
**3**	M	46	RUL	SBRT
**4**	M	80	LUL	SBRT
**5**	M	67	RLL	CRT
**6**	M	82	LUL	CRT
**7**	M	74	RLL	CRT
**8**	M	71	RLL	CRT
**9**	M	45	RLL	CRT
**10**	F	61	RUL	CRT
**11**	F	56	RUL	SBRT
**12**	M	85	LUL	SBRT
**13**	M	83	RUL	CRT
**14**	M	45	RLL	CRT
**15**	M	65	LUL	CRT
**16**	M	78	RUL	CRT
**17**	M	61	LUL	CRT
**18**	M	76	RUL	SBRT
**19**	M	69	LLL	CRT
**20**	F	72	LUL	SBRT
**21**	F	51	RUL, LUL	SBRT
**22**	M	74	RUL	CRT
**23**	M	71	RUL	CRT

RUL = right upper lobe; RLL = right lower lobe; LUL = left upper lobe; LLL = left lower lobe; CRT = conventional radiotherapy; SBRT = stereotactic body radiotherapy.

### Real-time position management (RPM) respiratory-gating system

The RPM respiratory-gating system (Varian Medical Systems, Palo Alto, CA, USA) was used to record breathing signals. Fig 1 shows the setup of the RPM system with a visible guiding system recording a patient’s respiration. The red bar graph at the bottom depicts changes to a phase with time. The patient in Fig 1 maintained a comfortable posture with the lower part of the body immobilized by the CIVCO Body Pro-Lok™ System (CIVCO Medical Solutions, Coralville, IA, USA). An external marker block (an infrared reflector) was placed on the abdomen, where the breathing of the patient could be clearly observed. An infrared camera recorded the movement of the external marker block as the patient breathed. The patient breathed while following the change in the graph with time by using the visible guiding system placed overhead in an attempt to increase the reproducibility of breathing.

The external marker block reflected infrared light as the patient breathed, while the infrared camera recorded the position of the external marker block in real-time by detecting the reflected infrared light to measure the breathing signals of the patient (Fig 1).

The RPM measurement system has two modes for measuring breathing signals: the amplitude mode based on the position and phase mode based on the cycle. Our study used only the phase mode, which is more widely used than the amplitude mode. The phase mode produces a sine-wave curve as the external marker block moves up and down over time, as shown in Fig 1.

### Acquiring respiration signals

Respiration signals obtained without the visible guiding system were free respiration signals, and those obtained after training with the visible guiding system were guided respiration signals. After the patient was placed on the Body Pro-Lok™ system and the Vac-Lok™ cushion was customized for the patient, the free respiration signal was recorded for 5 min. After the free respiration signal was obtained, patients were given an explanation about the visible guiding system and then trained to breathe in a stable manner while following the changing bar graph according to the recorded time in the portable media player (PMP). The breathing cycle was set to 3 s according to the breathing training protocol of the hospital. Although the breathing signals were recorded for 5 min, the data during the initial 3 min for free respiration and guided respiration were excluded from the analysis of the results because the patients were adapting to the visible guiding system during this time.

### Analysis of the respiration signals

The records of the breathing signals were saved as a data file that could be opened and read in a text editor such as Notepad++, and all of the recorded information could be identified. The recorded phase values were converted from radians to degrees. The position values of the external marker that corresponded to the phases 0%, 10%, 20%, 30%, 40%, 50%, 60%, 70%, 80%, and 90% were extracted.

**Fig 2:**
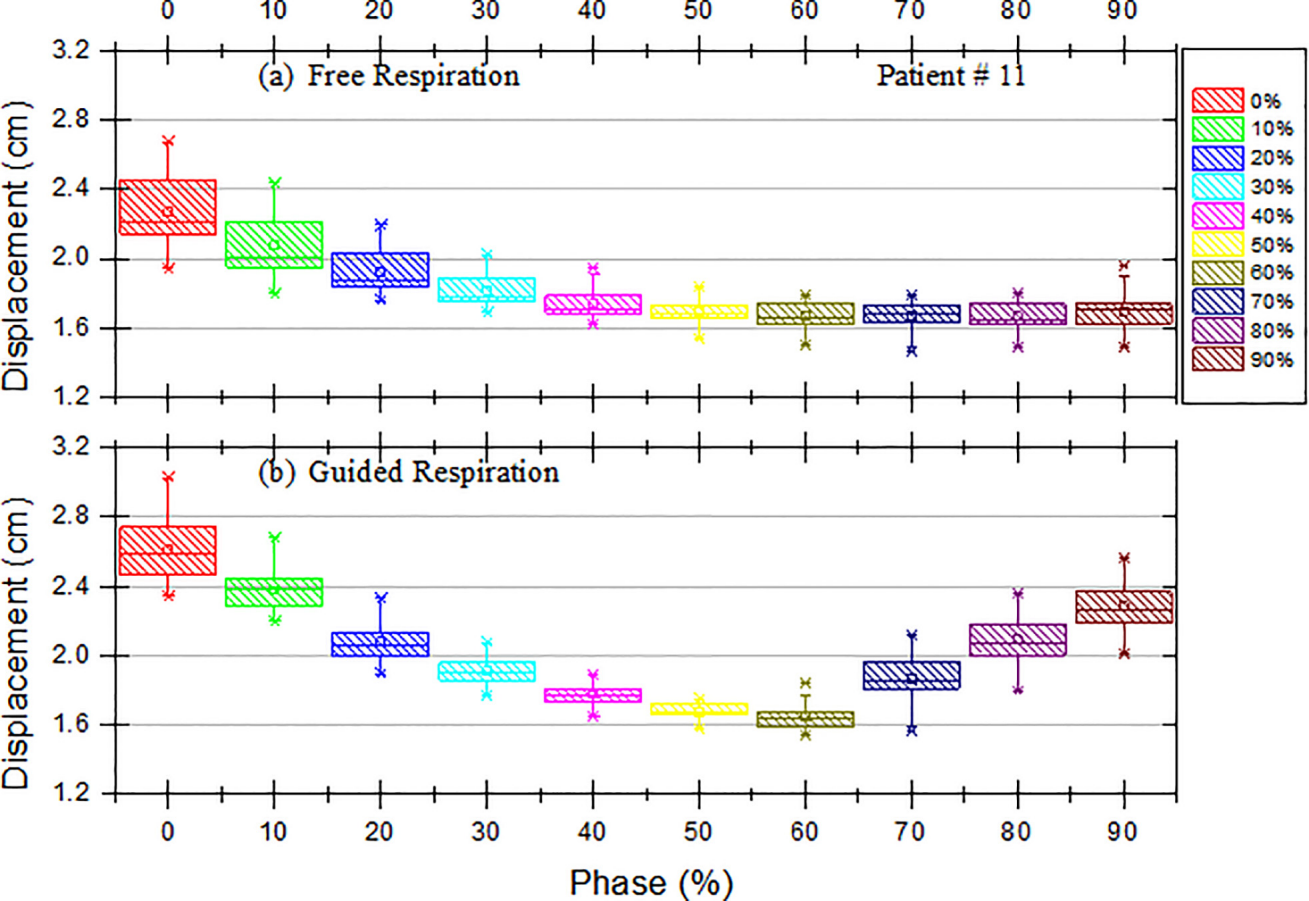
**Box and Whisker Plots of the Displacement of the External Block Marker as a Function of Phase for Patient #11 with (a) Free Respiration and (b) Guided Respiration.** The box represents the interquartile range of the results: the bottom line indicates the 25th percentile, the top line indicates the 75th percentile, the solid line inside the box represents the median, and the asterisk in the box represents the mean. The whiskers on the box represent a confidence interval of approximately 95%, and the points beyond the whiskers are extreme values outside the 95% confidence interval of the median.

**Fig 3:**
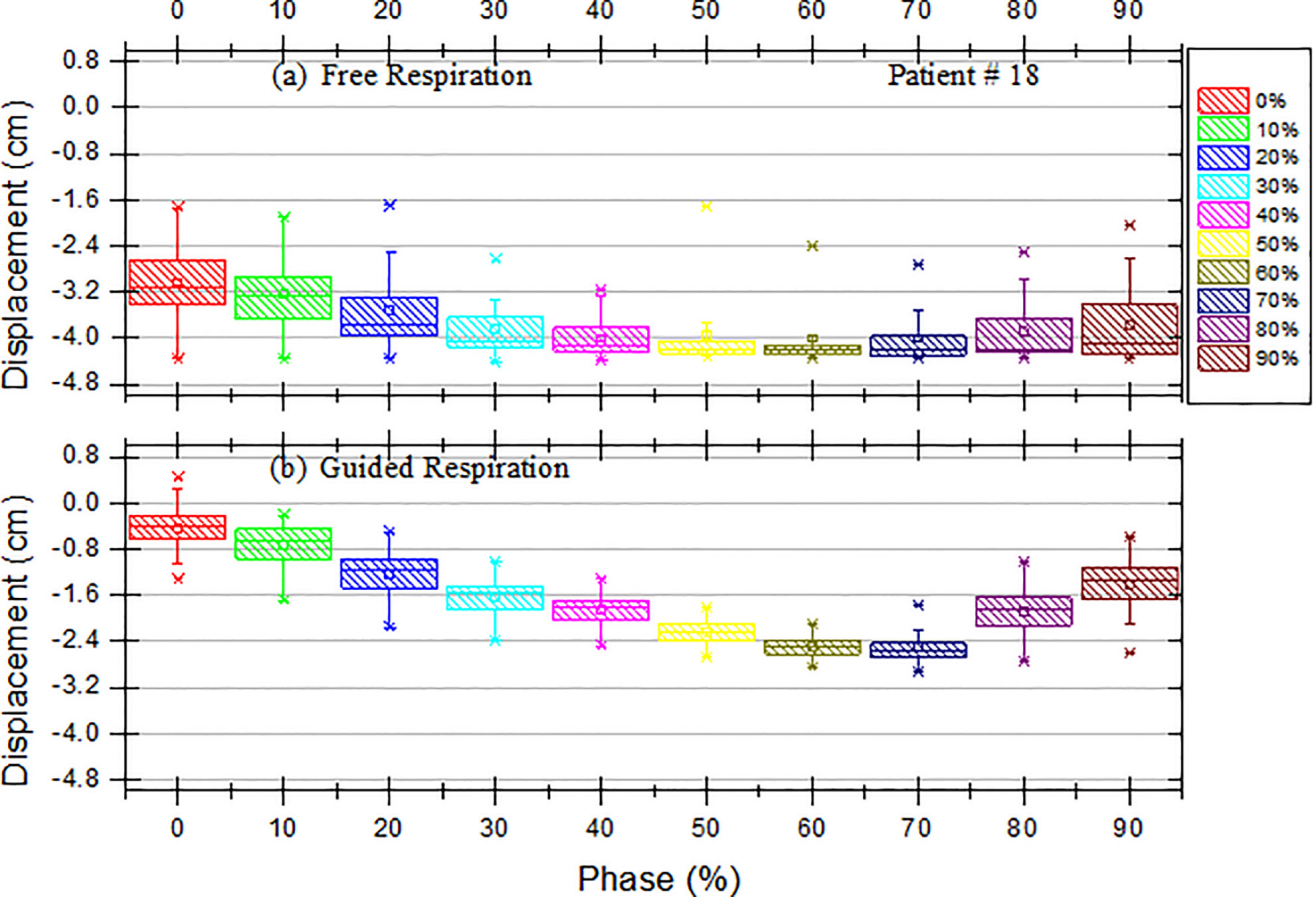
**Box and Whisker Plots of the Displacement of the External Block Marker as a Function of Phase for Patient #18 with (a) Free Respiration and (b) Guided Respiration.** The box represents the interquartile range of the results: the bottom line indicates the 25th percentile, the top line indicates the 75th percentile, the solid line inside the box represents the median, and the asterisk in the box represents the mean. The whiskers on the box represent a confidence interval of approximately 95%, and the points beyond the whiskers are extreme values outside the 95% confidence interval of the median.

The differences between the extracted position values recorded with and without the use of the visible guiding system were analyzed by using a graph showing the positions of the marker block over time and a box plot for each phase, as shown in Figs 2 and 3. In addition, a significant-difference analysis of the use of the visible guiding system was performed for each patient by using a paired *t*-test (SPSS, Inc., Chicago, IL, USA). After the analysis of the breathing signal from the guided respiration, we analyzed the standard deviation of the respiration signals for the 23 patients to find the optimal gating window with the visual guidance system. In this study, we restricted the meaning of “optimal” to the lowest standard deviation of the respiration signals with the external marker.

## Results

**Fig 4:**
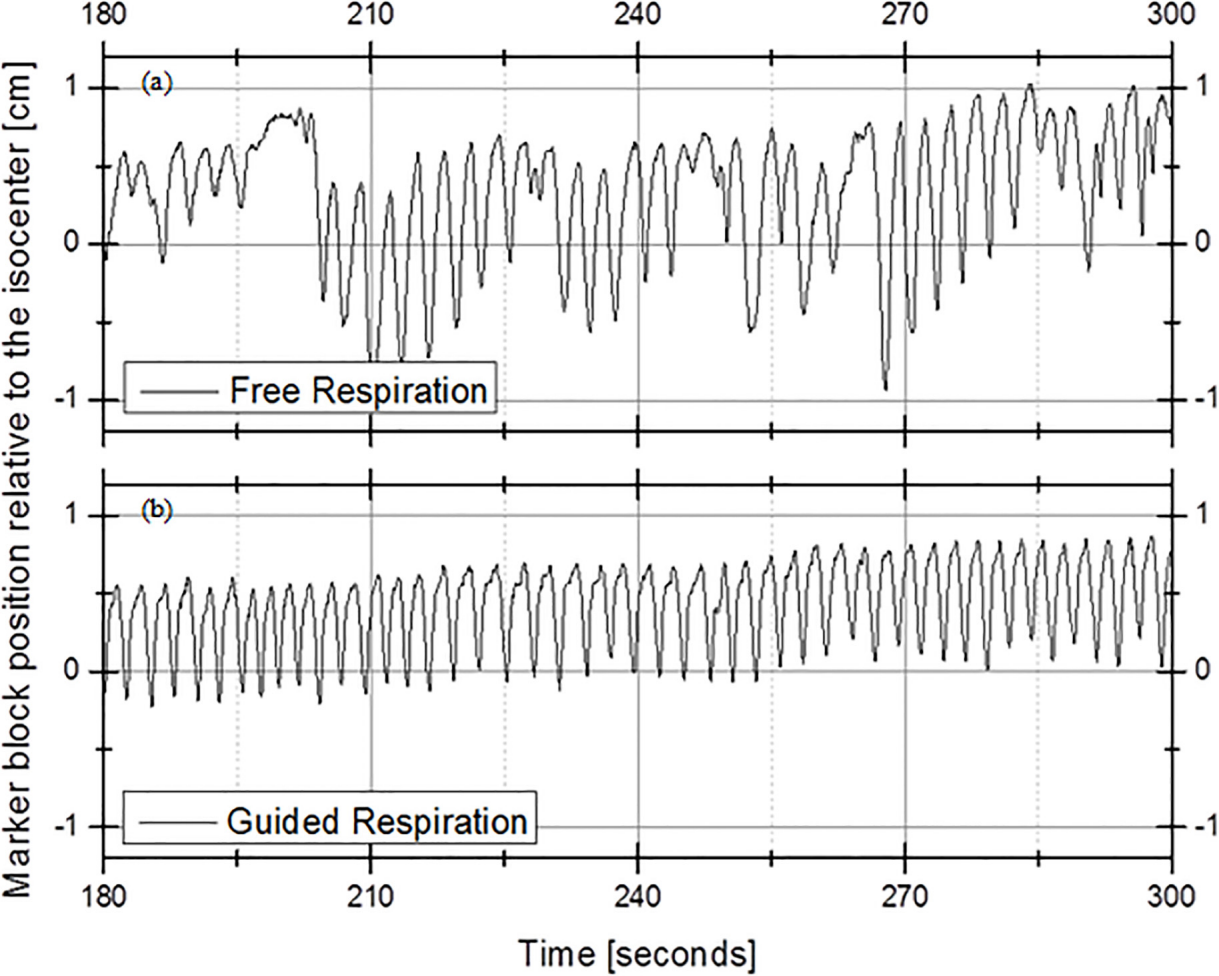
Amplitude of the Marker-block Position of Patient #13 as a Function of Time: (a) Free Respiration and (b) Guided Respiration.

Fig 4 shows the amplitude of the marker block position of Patient #13 as a function of time for (a) free respiration without the visible guiding system and (b) guided respiration with the visible guiding system. The *x* axis represents time (in seconds) from 3 min to 5 min from the beginning of the recording, and the *y* axis represents the position of the external marker block. The free respiration of patient #13 showed an irregular cycle from 3 min to 5 min and an irregular position of the marker block over time, as shown in Fig 4(A). On the other hand, Fig 4(B) shows the result for guided respiration with a regular cycle from 3 min to 5 min, and the position of the marker block was more regular relative to that for free respiration.

Fig 2 presents box and whisker plots for the displacement of the external block marker as a function of phase for Patient #11. The *x* axis represents the phase, and the *y* axis represents the displacement of the external marker block. Each box represents the interquartile range of the results: the bottom line indicates the 25th percentile, the top line indicates the 75th percentile, the solid line inside the box represents the median, and the asterisk represents the mean. The whiskers represent an approximately 95% confidence interval, and the points beyond the whiskers are extreme values outside the 95% confidence interval of the median. Fig 2(B) shows that the displacement of the marker block decreased with the phases 0%, 10%, 20%, 30%, 40%, 50%, and 60% for guided respiration compared with that for free respiration, as shown in Fig 2(A). This tendency was also found in Patient #18.

Table 2 lists the mean and standard deviation of the duration of each respiration cycle. The patients underwent breathing training according to the breathing-training protocol of Yeungnam University Medical Center with the breathing cycle set to 3 s. In addition, the table presents the mean value and standard deviation of the differences derived using an SPSS paired *t*-test, standard error, 95% confidence interval (lower limit, upper limit), and *p*-values. Differences with *p* < 0.05 were considered significant and are indicated with a superscript “a” on the *p-*value. The statistical results showed significant differences in breathing due to breathing training in 19 of the 23 patients. There were no significant differences in values due to breathing training for Patient #1 (*p* = 0.118), Patient #10 (*p* = 0.069), Patient #14 (*p* = 0.124), and Patient #19 (*p* = 0.374). We believe that this is because their breathing was stable even before breathing training.

**Table 2:** Results of the Paired *t*-test for Differences between Free Respiration and Guided Respiration with the 3-s Cycle Using the Visual Guidance System (*N* = 40 with Respiration Signals).

Patient	Respiration	Mean±SD (s)	Difference between paired data				*p*-value of paired *t*-test
			Mean±SD (s)	Standard error (s)	95% CI		
					Lower limit	Upper limit	
**Patient#1**	Free	3.393±1.160	0.314±1.242	0.196	-0.084	0.711	0.118
	Guided	3.080±0.955					
**Patient#2**	Free	3.451±1.313	0.454±1.400	0.221	0.007	0.901	0.047^a^
	Guided	2.997±0.682					
**Patient#3**	Free	3.851±1.485	0.706±1.805	0.285	0.129	1.283	0.018^a^
	Guided	3.145±0.866					
**Patient#4**	Free	3.693±1.134	0.685±1.302	0.206	0.269	1.101	0.002^a^
	Guided	3.008±0.663					
**Patient#5**	Free	3.755±0.997	0.607±1.368	0.216	0.169	1.044	0.008^a^
	Guided	3.148±0.939					
**Patient#6**	Free	3.647±0.988	0.742±1.187	0.188	0.363	1.122	<0.001^a^
	Guided	2.905±0.648					
**Patient#7**	Free	3.752±0.807	0.621±1.392	0.220	0.176	1.066	0.007^a^
	Guided	3.132±1.115					
**Patient#8**	Free	4.650±2.076	1.165±2.407	0.381	0.395	1.934	0.004^a^
	Guided	3.485±1.547					
**Patient#9**	Free	4.969±2.196	1.680±2.184	0.345	0.982	2.379	<0.001^a^
	Guided	3.289±0.755					
**Patient#10**	Free	3.462±0.814	0.313±1.057	0.167	-0.025	0.651	0.069
	Guided	3.149±0.670					
**Patient#11**	Free	5.267±1.364	2.200±1.517	0.240	1.715	2.685	<0.001^a^
	Guided	3.067±0.835					
**Patient#12**	Free	4.117±1.600	0.770±1.898	0.300	0.162	1.377	0.014^a^
	Guided	3.348±1.222					
**Patient#13**	Free	3.745±1.461	0.752±1.504	0.238	0.272	1.233	0.003
	Guided	2.993±0.151					
**Patient#14**	Free	2.973±0.554	-0.250±1.003	0.159	-0.570	0.072	0.124
	Guided	3.222±0.771					
**Patient#15**	Free	4.268±1.725	1.034±2.068	0.327	0.373	1.700	0.003^a^
	Guided	3.234±0.805					
**Patient#16**	Free	3.866±1.566	0.873±1.608	0.254	0.359	1.388	0.001^a^
	Guided	2.996±0.316					
**Patient#17**	Free	3.581±0.941	0.587±1.149	0.182	0.220	0.955	0.002^a^
	Guided	2.994±0.526					
**Patient#18**	Free	5.403±1.717	2.328±2.021	0.320	1.681	2.974	<0.001^a^
	Guided	3.076±0.749					
**Patient#19**	Free	2.876±1.113	-0.182±1.284	0.203	-0.593	0.228	0.374
	Guided	3.059±0.759					
**Patient#20**	Free	3.972±1.277	0.900±1.430	0.226	0.442	1.357	<0.001^a^
	Guided	3.072±0.569					
**Patient#21**	Free	4.474±1.310	1.472±1.374	0.217	1.033	1.912	<0.001^a^
	Guided	3.001±0.189					
**Patient#22**	Free	3.990±0.847	0.991±0.833	0.132	0.724	1.258	<0.001^a^
	Guided	2.999±0.148					
**Patient#23**	Free	3.861±0.623	0.861±0.628	0.099	0.660	1.062	<0.001^a^
	Guided	3.000±0.195					

CI = confidence interval.

^a^*p* < 0.05.

Table 3 presents the means of the standard deviations of each phase after the analysis of the guided-respiration breathing signals of the 23 patients. The standard deviation was calculated from the motion amplitudes for each phase. The phases showed the lowest difference in standard deviation in the range of 30%–70%.

**Table 3:** Results of the Standard Deviation of the Respiration Signals for the 23 Patients Using the Visual Guidance System.

Phase	Mean (range)
**0%**	0.300 (0.120–0.672)
**10%**	0.281 (0.102–0.567)
**20%**	0.246 (0.092–0.488)
**30%**	0.192 (0.066–0.470)
**40%**	0.162 (0.046–0.483)
**50%**	0.143 (0.029–0.537)
**60%**	0.156 (0.039–0.500)
**70%**	0.218 (0.048–0.532)
**80%**	0.258 (0.085–0.563)
**90%**	0.277 (0.099–0.614)

## Discussion

The introduction of the four-dimensional treatment planning system led to the study of the dynamic motion of tumors in mobile lungs [14–18]. Several methods have been proposed to minimize the errors caused by the irregular breathing of patients, including the (1) motion-encompassing method, (2) respiratory-gated techniques, (3) breath-hold techniques, (4) forced shallow-breathing methods, and (5) respiration-synchronized techniques [13].

In our study, the differences in breathing due to breathing training using the visible guiding system were presented as a function of time and the position of the external marker block. The study was limited in that only the vertical distance relative to the iso-center horizontal could be determined with the external marker block, not the lateral and longitudinal distances in the translational directions. In fact, the external marker does not represent the actual three-dimensional movement of the tumor, but Beddar et al. [19] indicated that there is a strong correlation between the motions of the external marker and internal fiducial marker and suggested that the marker block of the RPM system is a reliable predictor. Because we used a one-dimensional RPM system (anterior–posterior), which is still used in many hospitals to determine the gating window in gated radiotherapy, the results of this study should help the many institutions that use one-dimensional RPM systems.

We assumed that the correlation between the external marker and internal tumor remains constant in the vertical direction. Statistical analysis was performed by using the paired *t*-test on a 3-s cycle for cases with and without breathing training. As shown in Fig 4, for free respiration without the use of the visible guiding system, both the cycle over time and the positions of the external marker block had irregular distributions.

Berbeco et al. [20] assessed the residual tumor motion for the amplitude-based gating mode and phase-based gating mode. They pointed out that each mode can be problematic if a patient’s depth of breathing is inconsistent and the patient’s respiration signals are affected by a baseline shift. This can cause the beam to turn on during an unintended gating time.

Similar to the results of our study, Jiang [16] demonstrated differences between free breathing and coached breathing depending on whether or not a coaching technique was used. He used the initial recording for approximately 200 s and showed that the cycle over time and the displacement of the external marker clearly had smaller variations with coached breathing than with free breathing. These are similar to the results of our study. In addition, Jiang discussed the Mitsubishi/Hokkaido technique for internal gating and the Massachusetts General Hospital (MGH) technique for external gating. He noted that the gating window of non-gated 3D conformal radiation therapy (CRT) was 100%, whereas that of typical gated 3DCRT treatment was 30%–50%. Furthermore, he explained that if the delivery technique for gated IMRT is step-and-shoot, less than 30% of the gating window would be better owing to the beam-off time needed for multi-leaf collimator (MLC) motion.

Vedam et al. [18] reported a trend toward an increase in standard deviation and range of motion with an increase in the gating window. In addition, they found that the use of a breathing coaching method could improve the range and standard deviation of motion depending on the function of the gating window; however, these results could differ depending on the patient.

Berbeco et al. [15] reported that a number of clinics began performing gated radiotherapy by using an external marker to reduce the amount of radiation on healthy lung tissues. Because the end-of-exhale (EOE) tumor position is more reproducible than that during the rest of the breathing cycle, the gating window was positioned at the exhale. On the other hand, because the expanded lungs reduce the amount of healthy tissue within the treatment field, the end-of-inhale (EOI) phase would be more beneficial. In phase-based gating, the mean values of the residual motion were 2.7 mm at the EOI phase and 1.2 mm at the EOE phase, which implies that the residual motion of the tumors was larger in the EOI phase than in the EOE phase.

In our study, statistical analysis of the breathing signals of the 23 patients who underwent breathing training showed that a gating window of 40% was considered optimal, and the 30%–70% gating window was considered most reasonable for use in guided respiration treatment. We limited the meaning of “optimal” to the reproducibility of the external marker. If the 30%–70% phase is used for the gating window of the visible guiding system with RGRT, the unnecessary loss of time from irregular breathing would be minimized, and the effectiveness of the treatment would be increased.

## Conclusions

In this study, a statistical analysis was conducted on breathing signals obtained before and after breathing training with a visible guiding system. The results showed that the repeatability and reproducibility of the breathing cycle improved with breathing training and that the optimal gating window for RGRT is 40% (30%–70%) with respect to repeatability for breathing after respiration training with the visible guiding system.
